# A compilation of reported alterations in the cerebrospinal fluid proteome in Alzheimer's disease

**DOI:** 10.1093/braincomms/fcaf202

**Published:** 2025-05-23

**Authors:** Matthijs B de Geus, Angus C Nairn, Steven E Arnold, Becky C Carlyle

**Affiliations:** Department of Neurology, Massachusetts General Hospital, Charlestown, MA 02129, USA; Cell & Chemical Biology, Leiden University Medical Center, 2333ZC Leiden, The Netherlands; Department of Psychiatry, Yale University, New Haven, CT 06511, USA; Department of Neurology, Massachusetts General Hospital, Charlestown, MA 02129, USA; Neurology, Harvard Medical School, Boston, MA 02115, USA; Department of Physiology Anatomy and Genetics, Oxford University, Oxford OX1 3PT, UK; Kavli Institute for Nanoscience Discovery, Oxford OX1 3QU, UK

**Keywords:** CSF, systematic review, biomarkers, Alzheimer’s disease, proteomics

## Abstract

Alzheimer's disease is a multifaceted neurodegenerative disorder, with diverse underlying pathophysiological processes extending beyond amyloid-β and tau accumulation. The heterogeneity of Alzheimer's disease necessitates the identification of a broad array of biomarkers that capture the diverse mechanisms contributing to disease onset and progression. In this study, we systematically compiled and analysed cerebrospinal fluid proteomics data from omics studies utilizing mass spectrometry, Olink, or SomaScan platforms. Systematic literature searches for each platform revealed a total of 264 studies. From this, a set of 18 studies were selected based on sample size, number of markers analysed, and open data availability. We found a total of 1,448 differentially expressed proteins between Alzheimer's disease and amyloid negative controls across these datasets, with 635 being found in more than one study. A ‘top’ set of 61 differentially expressed proteins were consistently reported in at least six studies. Clustering and functional enrichment analysis of the top differentially expressed proteins indicated involvement in metabolic regulation, glutathione metabolism and proteins of the 14-3-3 family, reflecting importance of reactive oxygen species (ROS) response. Synaptic signalling processes were found to generally be downregulated. We further integrated the top differentially expressed proteins with results from a study on familial Alzheimer's disease cerebrospinal fluid to assess at which stage of disease progression these proteins change, highlighting markers shared between sporadic and familial Alzheimer's disease datasets. Lastly, we examine the overlap of the top differentially expressed proteins between cerebrospinal fluid and brain tissue using a publicly available database. This analysis provides a comprehensive overview of the Alzheimer's disease cerebrospinal fluid proteomic landscape, indicating changes in key pathways and cellular processes associated with Alzheimer's disease pathology. By integrating data from different platforms, we highlight reproducible protein changes that may serve as promising candidates for further biomarker research aimed at improving patient stratification, tracking disease progression, and assessing therapeutic interventions.

## Teaser

de Geus *et al*. report that a compilation of CSF proteomics studies in Alzheimer’s disease identified 61 consistently altered proteins across mass spectrometry, Olink and SomaScan platforms. The findings highlight disease heterogeneity, with changes in glycolytic metabolism, response to oxidative stress and synaptic signalling, suggesting potential biomarkers for disease progression.

## Graphical Abstract

**Graphical Abstract:**
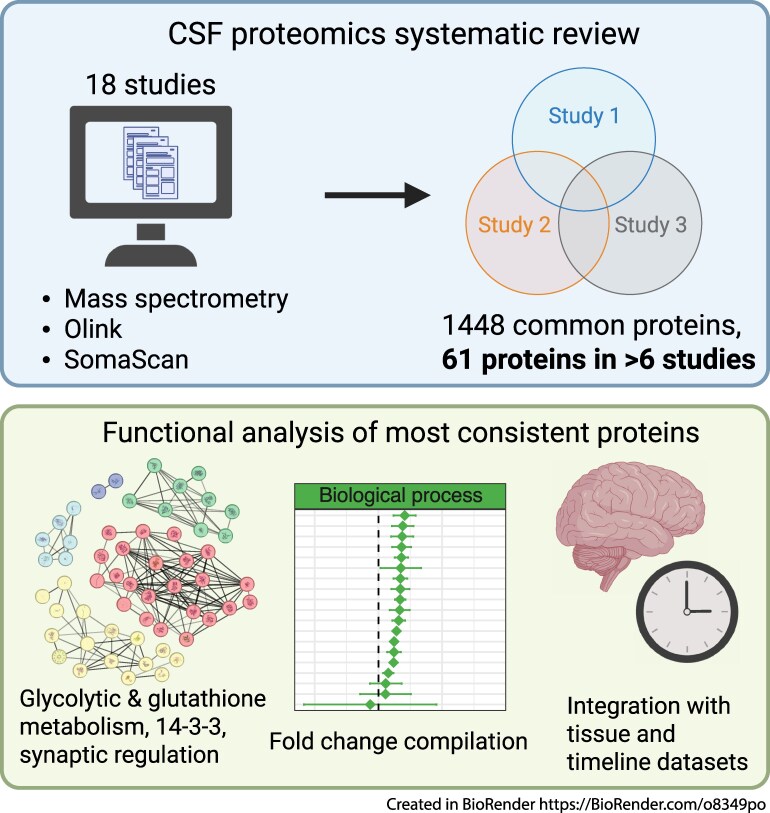

## Introduction

Alzheimer's disease is the most common form of dementia, characterized by progressive cognitive decline and neurodegeneration. The core pathologies include the formation of extracellular amyloid-ß plaques and intracellular neurofibrillary tangles of phosphorylated tau. Although these components have been extensively studied, they fail to fully capture the multifaceted pathophysiological processes underlying Alzheimer's disease.^1,2^ These features may contribute differently between patient subpopulations as well as at different stages of disease progression.^3,4^ Establishing biomarkers of these contributions is needed to improve patient diagnosis, treatment discovery and development and tracking disease progression.

Mass spectrometry (MS)-based methods have driven the exploration of the cerebrospinal fluid (CSF) proteome in Alzheimer's disease over the past decade, enabling large-scale screening of biofluids in both unbiased and targeted ways to identify biomarkers and better understand disease-related processes.^5-18^ Advances in throughput, study design and robustness have enabled the investigation of larger cohorts and more accurate measurements of analytes.^19,20^ Over the past few years, multiplex affinity-based platforms, such as Olink and SomaScan, also have seen increasing use to study changes in the CSF proteome.^21-24^ The Olink platform is based on proximity extension assay technology, which uses DNA-tagged target-specific antibodies. Upon binding to their target, quantitative PCR or next-generation sequencing allow for relative protein quantification.^25^ The SomaScan platform uses DNA-based aptamers (SOMAmers) that are target specific due to their unique three-dimensional structure. The SOMAmers can then be quantified using next-generation sequencing to measure relative protein levels in the sample.^26^ These affinity-based platforms provide relative quantifications with a high sensitivity, broad dynamic range and high-throughput capabilities, and require less technological expertise to operate than MS platforms.^27^ Compilation of results across the different platforms is needed to enable cross-validation of findings for greater confidence in the identified biomarkers or mechanistic insights. There have been a handful of other meta-analyses that investigated the proteomic changes in Alzheimer's disease CSF,^20,28-30^ but none have combined MS- and affinity-based studies.

In this study, we present a compilation of CSF proteomics studies in Alzheimer's disease, including both MS- and affinity-based techniques. First, we performed a systematic literature search to identify Alzheimer's disease proteomics studies in CSF that analysed at least 40 different proteins across a minimum of 10 participants. Then, we determined the most commonly changed proteins across studies, and analysed the underlying biological processes (BPs) associated with Alzheimer's disease. We then compiled the reported fold-change (FC) data across studies to determine the consistency of changes across those BPs. We compare our data with a study on autosomal dominant Alzheimer's disease^4^ to show how these proteins change over the course of disease progression in familial Alzheimer's disease. Finally, comparison with a publicly available repository of brain tissue proteomics highlights the commonalities between changes in CSF and brain tissue in Alzheimer's disease.

## Materials and methods

### Search strategy and study selection

**Figure 1:**
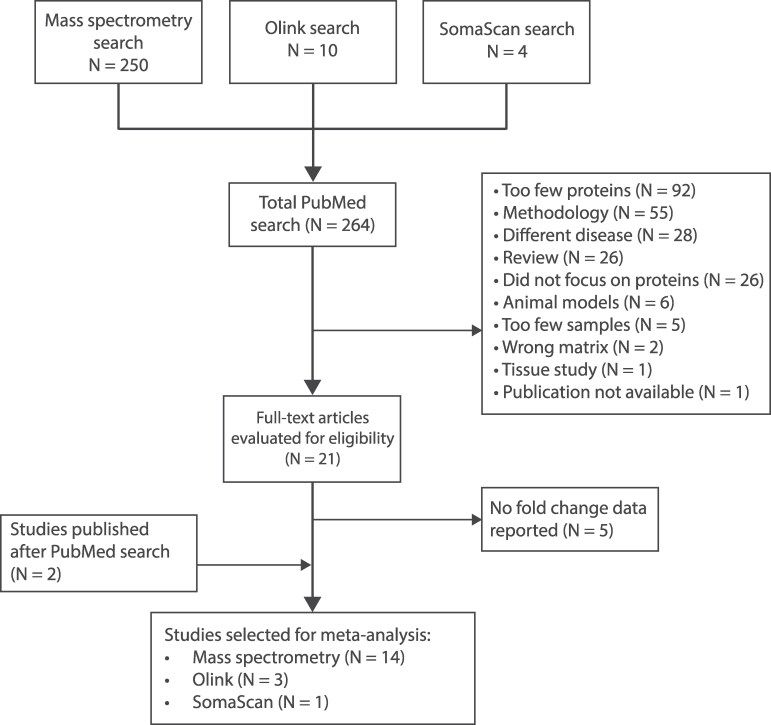
**Flow diagram of systematic literature search and inclusion in the meta-analysis.** Three separate searches were performed for studies using MS, Olink and SomaScan based screening techniques. In total, this resulted in 264 hits on PubMed. After applying exclusion criteria, 21 studies were fully evaluated for eligibility. Five studies did not report any fold-change data and were excluded from our analyses. Two studies that were published after the initial literature search were included. In total, 18 studies were used in our meta-analysis. Three studies reported multiple cohort analyses, which were included as separate comparisons resulting in a total of 21 comparisons.

Studies were searched on PubMed on 12 June 2024. Three separate searches were performed for studies using either MS, Olink or SomaScan (Fig. 1). The following search terms were used for MS studies: ‘Alzheimer's disease AND (proteomics OR biomarker) AND cerebrospinal fluid AND (‘mass spectrometry’) NOT plasma’. For studies using Olink methods the search term ‘mass spectrometry’ was replaced with (‘proximity extension assay’ OR ‘PEA’ OR ‘Olink’), and for studies using SomaScan methods replaced with (‘Somalogic’ OR ‘Somascan’). The PubMed filters for English and Human participants were selected and a timeframe from 2014 to 2024 was set. After the initial search on PubMed, all studies were exported and manually screened by author MDG. For the purpose of this study, we only included studies that had a minimum of 10 participants and quantified a minimum of 40 proteins. We limited our analysis further to studies that openly provided accessible and fully available data to ensure transparency and reproducibility in the analysis.

### Data preparation

Data from all studies were manually extracted from manuscripts and supplementary data. Studies used varying ways of defining their Alzheimer's disease and control cohorts, based on either clinical tests, fluid biomarker tests of Aβ, imaging methods, or a combination. For our meta-analysis the individual study-defined Alzheimer's disease (Aß+) versus control contrasts (Aß−) were used. The data collected from the studies included lists of all the proteins quantified by MS or assayed with Olink or SomaScan, as well as lists of significantly changed proteins between contrasts and where available, the accompanying FC data (Table 1). Significant proteins were defined by the false-discovery rate (FDR) cut-off used in each respective study. If FC data were not reported but sufficient raw data were available, log2 FC values were calculated, and *P*-values were determined using unpaired two-sided *t*-tests and FDR corrected using Benjamini–Hochberg *P*-value adjustment. To allow for direct comparisons between the studies, the datasets were manually adjusted using a set of steps. A single gene name was generated corresponding to the IDs used in the studies. If protein groups or multiple names were reported, only the first ID was selected. IDs were stripped of isoforms. All data were handled in R (version 4.3.1) and Microsoft Excel. Data were visualized using Tidyverse packages (version 2.0.0), ggplot2 (version 3.5.1), ggpubr (version 0.6.0) and ggrepel (version 0.9.3). UpSet plots of overlapping proteins between studies were visualized using the *UpSetR* package (version 1.4.0). Figures were prepared using Adobe Illustrator (version 27.2).

**Table 1:** Overview of studies included in meta-analysis

Author	DOI	Year	Sub-cohort	Technique	Sample size	# Total proteins measured	# Significant proteins	Included in FC analysis
*Spellman et al.^5^*	10.1002/prca.201400178	2015		MRM-MS	Alzheimer's disease (66) versus Control (85)	142	0	Yes
*Dayon et al.^6^*	10.1186/s13195-018-0397-4	2018		TMT-MS	Alzheimer's disease (72) versus Control (48)	790	22	No
*Sathe et al.^7^*	10.1002/prca.201800105	2019		TMT-MS	Alzheimer's disease (5) versus Control (5)	2327	139	Yes
*Bader et al.^8^*	10.15252/msb.20199356	2020	Sweden cohort	DIA-MS	Alzheimer's disease (29) versus Control (31)	1484	540	Yes
			Magdeburg-Kiel cohort	DIA-MS	Alzheimer's disease (26) versus Control (12)	1484	453	Yes
*Higginbotham et al.^9^*	10.1126/sciadv.aaz9360	2020		TMT-MS	Alzheimer's disease (20) versus Control (20)	2875	528	Yes
*Bai et al.^10^*	10.1016/j.neuron.2019.12.015	2020		TMT-MS	Alzheimer's disease (8) versus Control (5)	5940	9	Yes
*Johnson et al.^11^*	10.1038/s41591-020-0815-6	2020		TMT-MS	Alzheimer's disease (147) versus Control (150)	532	59	Yes
*Park et al.^12^*	10.1038/s41598-020-64461-y	2020		SWATH-MS	Alzheimer's disease (42) versus Control (39)	274	21	Yes
*De Geus et al.^13^*	10.1038/s41598-023-49440-3	2023		DIA-MS	Alzheimer's disease (72) versus Control (68)	636	101	Yes
*Haque et al.^14^*	10.1126/scitranslmed.adg4122	2023		SRM-MS	Alzheimer's disease (110) versus Control (376)	48	22	Yes
*Liu et al.^15^*	10.3390/ijms241814225	2023		DDA-MS	Alzheimer's disease (12) versus Control (10)	1308	68	No
*Modeste et al.^16^*	10.1186/s13024-023-00638-z	2023	Caucasian sub-cohort	TMT-MS	Alzheimer's disease (53) versus Control (47)	1840	257	Yes
			African American sub-cohort	TMT-MS	Alzheimer's disease (52) versus Control (51)	1840	313	Yes
*Watson et al.^17^*	10.1038/s41597-023-02158-3	2023		SRM-MS	Alzheimer's disease (130) versus Control (130)	51	30	Yes
*Del Campo et al.^21^*	10.1038/s41467-023-41122-y	2023		Olink	Alzheimer's disease (235) versus Control (190)	665	14	No
*Kamalian et al.^23^*	10.3390/biom13071094	2023		Olink	Alzheimer's disease (38) versus Control (48)	2936	117	No
*Tijms et al.^18^*	10.1038/s43587-023-00550-7	2024		TMT-MS	Alzheimer's disease (419) versus Control (187)	1309	419	No
*Pichet Binette et al.^22^*	10.1038/s41593-024-01737-w	2024		Olink	Alzheimer's disease (184) versus Control (352)	1331	51	No
*Guo et al.^24^*	10.1038/s41562-024-01924-6	2024	Biologically defined Alzheimer's disease	SomaScan	Alzheimer's disease (269) versus Control (85)	6361	279	No
			Clinically defined Alzheimer's disease	SomaScan	Alzheimer's disease (138) versus Control (166)	6361	50	No

### Biological enrichment analysis

Biological enrichment analysis was performed using the STRING-db platform^31^ (v12.0). The top 61 proteins, overlapping between 6 or more studies, were used as input for the ‘multiple proteins search’ option with the ‘Homo sapiens’ organism parameter. Two of the top differentially expressed proteins (DEPs), lactate dehydrogenase B (LDHB) and malate dehydrogenase 1 (MDH1), were not identified on the STRING database and therefore not included in the enrichment analysis. A background list of 6704 unique protein names was generated for the enrichment analysis, based on all unique proteins identified across all the MS studies. Proteins were clustered using a k-means approach with nine clusters. Clusters were functionally annotated according to their most enriched gene ontology (GO) BP terms. For clusters that were annotated with multiple terms, one was chosen for visualization purposes, and all annotations can be found in Supplementary Table 1. Functional enrichment visualization of BP terms was performed using the STRING-db function against the specified background with the following parameters: group term similarity was set to 0.8 or higher, terms were sorted by signal with a minimum of 0.01, a minimum of two proteins per network was set, a minimum strength of 0.01 and a maximum FDR of 0.05.

### Fold-change analysis

Inclusion in the FC analysis was limited to studies that reported FC values or provided sufficient data for FC calculation. Studies using Olink did not report FCs and the SomaScan study reported an unspecified FC score ‘beta’, not translatable to a FC score comparable with the other studies. All reported FCs were transformed to the log2 space to allow for direct comparison between studies. Directions of FC were harmonized across studies to make positive FC represent upregulation in Alzheimer's disease. Average FC and 95% confidence interval (CI) were calculated for each top protein across all the studies and it was quantified, regardless of significance. FCs were visualized using forest plots.

### Integration with onset of familial Alzheimer’s disease dataset

Top overlapping proteins were compared with biomarker data from Johnson *et al*.^4^ Proteins were manually binned into four categories over the course of disease development in familial Alzheimer's disease as follows: ‘Early’ (25+ years before disease onset), ‘Mid’ (25–5 years before disease onset), ‘Onset’ (5 years before—5 years after disease onset) and ‘Late’ (5+ years after disease onset). Proteins were binned based on the first moment of increase/decrease of the marker, reported in Johnson *et al.* (2023).

### Comparison with tissue data

Protein data in tissue was extracted from the Neuropro database.^32^ Only proteins that were found to be consistently differentially expressed in at least 5 studies (Neuropro score > 5) were used to compare with the top DEP proteins in CSF, comprising a dataset of 848 proteins.

### Statistical analyses

All statistical analyses were performed using R (version 4.3.1). For studies where raw data were available but FC values were not reported, log_2_-transformed FCs and associated *P*-values were calculated using unpaired two-sided *t*-tests and resulting *P*-values were adjusted with the Benjamini–Hochberg method. Linear relationships between number of DEPs and sample size or number of proteins measured was assessed using the *lm()* function.

## Results

### Studies included in analysis

A total of 264 studies were found on PubMed across the three different search strategies (Fig. 1; Supplementary Table 2). Most studies were excluded for investigating too few analytes (*N* = 92; <40 analytes). Other reasons for exclusion were method development (*N* = 55), focusing on a non-Alzheimer's disease (*N* = 28), reviews (*N* = 26), analysing non-protein analytes (*N* = 26), animal model data (*N* = 6), too few samples (*N* = 5; <10 samples), analysing a different matrix (*N* = 2), studying tissue samples (*N* = 1) and the publication not being publicly accessible (*N* = 1). 21 publications were fully evaluated for their eligibility after which five MS studies were excluded, based on no openly accessible FC data being available. Two more studies published after the initial PubMed search was included in further analyses. In total 18 different studies were included for full analysis (Table 1). Three studies included analyses across different cohorts, which we included as separate comparisons, resulting in a total of 21 different datasets analysed. Fourteen studies used MS-based methods, 3 used Olink assays and 1 used SomaScan.

### Most consistent changes in Alzheimer’s disease cerebrospinal fluid proteome

**Figure 2:**
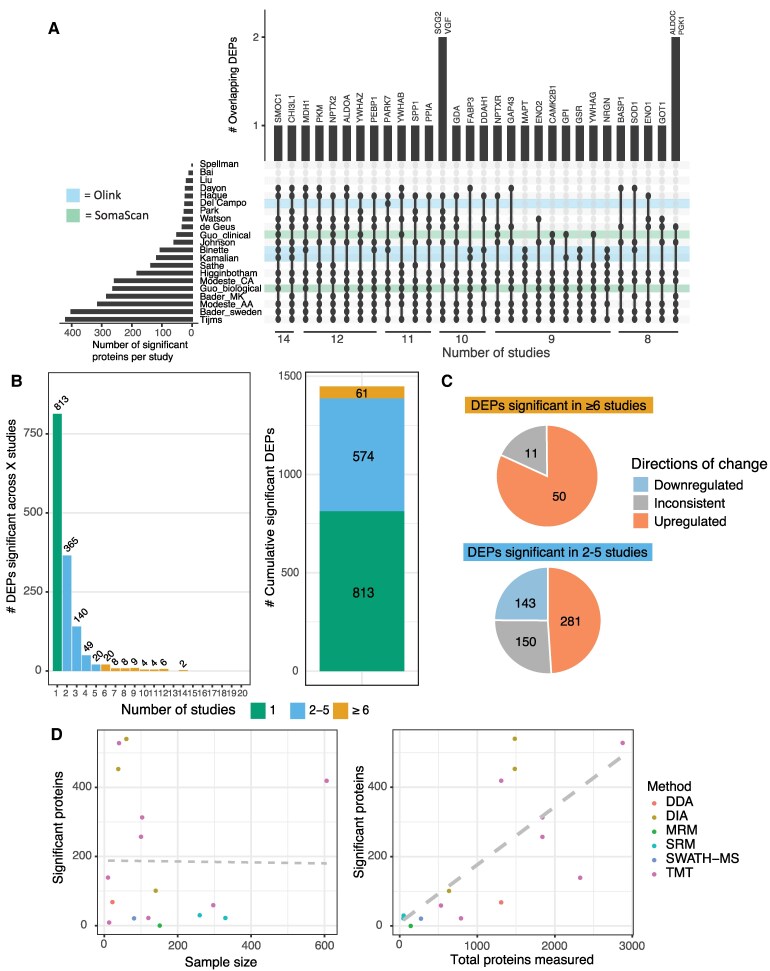
**DEPs identified across studies.** (**A**) Upset plot of the overlap of the 32 most frequently identified DEPs across all studies. Olink studies are highlighted in blue and SomaScan studies in green. The *x*-axis indicates the number of studies each protein was identified in. The *y*-axis shows the number of proteins in each overlap and the specific protein names are indicated above the bar-graph. (**B**) Distribution of DEPs across studies. 813 DEPs were significant in only one study (green bar), while 61 DEPs were consistently reported significant across six or more studies. (**C**) Of the top 61 overlapping significant DEPs, 50 were found to be consistently upregulated across studies. The proportion of DEPs with an inconsistent direction of change across studies was higher for DEPs that were found in two to six studies compared to the top overlapping DEPs. (**D**) Pearson correlation analysis revealed a significant association between the number of proteins measured after QC and the number of DEPs in MS studies (*r* = 0.722, *P* = 0.002). No significant correlation was observed between sample size and number of DEPs (*r* = −0.069, *P* = 0.808). Dots represent different studies and are coloured by MS method. DDA, data-independent acquisition; DIA, data-independent acquisition; MRM, multiple reaction monitoring; SRM, selected reaction monitoring; SWATH-MS, sequential window acquisition of all theoretical mass spectra; TMT, tandem mass tag labelled.

Across all studies there were 1448 significantly DEPs between Alzheimer's disease and controls. The full overlap of proteins across all studies is visualized in Supplementary Fig. 1 and the 32 proteins with the most overlap between studies are visualized in Fig. 2A. Two DEPs, ‘SPARC-related modular calcium-binding protein 1’ (SMOC1) and ‘chitinase-3 like-protein-1’ (CHI3L1, also known as YKL-40), both well-established markers of Alzheimer's disease,^33,34^ were found across 14 studies constituting the highest overlap among all studies (Supplementary Table 3). Other top overlapping proteins included ‘pyruvate kinase’ (PKM), ‘neuropentraxin 2’ (NPTX2), ‘aldolase A’ (ALDOA), ‘phosphatidylethanolamine binding protein 1’ (PEBP1), MDH1 and 14-3-3ζ (YWHAZ), which were found across 12 different studies. 813 DEPs (56%) were found in only 1 study, and 574 DEPs (39.6%) were found in 2 to 6 studies (Fig. 2B); 1010 DEPs were identified in MS-based studies only, whereas 286 DEPs were identified across both affinity-based platforms. The remaining 152 DEPs were identified across both MS and affinity-based platforms (Supplementary Table 4).

Of the 61 top DEPs significant in 6 or more studies, 50 were consistently upregulated in Alzheimer's disease compared to controls across all studies, with an inconsistent direction of change in the remaining 11 DEPs (Fig. 2C; Supplementary Table 5). For these 11 inconsistent top DEPs, there was a general consensus in the direction of change across studies with one or two indicating a different direction. But there was no pattern of specific studies that were the cause of the outliers. The inconsistent studies were all MS-based studies, with the SomaScan and Olink studies agreeing with the other MS-based studies. Four of these DEPs, ‘chromogranin B’ (CHGB), neuropeptide y (NPY), SCG2 and VGF are all post-translationally cleaved by proteases, resulting in differentially abundant peptide products, potentially giving rise to the inconsistencies between studies. Of the DEPs significant in 2 to 6 studies, 281 (48.9%) were consistently upregulated in Alzheimer's disease and 143 (24.9%) were consistently downregulated in Alzheimer's disease. The proportion of inconsistent proteins was higher in this group (150; 26.1%), compared to the top 61 DEPs. An overview of the platforms in which the top DEPs were identified can be found in Supplementary Table 6. Fifteen of the 61 top DEPs (24.6%) were identified across all three platforms, and 32 (52.5%) were identified across two platforms.

Exploratory analyses were performed to determine the effect of sample size or dataset size (number of proteins detected) on the number of DEPs. No significant correlations were found for either the sample size or total proteins measured with the number of DEPs across all types of study (Supplementary Fig. 2A). However, when looking only at MS studies (excluding one *n* = 13 study^10^), a significant correlation was found between total proteins measured and the number of DEPs (*P*-value = 0.002, *R*^2^ = 0.4848; Fig. 2D). No significant relationship was apparent between the number of proteins measured and the number of DEPs detected that were unique to their study (Supplementary Fig. 2B), although it is worth noting that studies quantifying >1000 proteins were more likely to have more than 25 unique DEPs than studies quantifying fewer than 1000. We also investigated the proportion of significant upregulated/downregulated proteins found in the different study types (MS, Olink and SomaScan; Supplementary Fig. 2C). All three methods showed a higher proportion of upregulated proteins than downregulated proteins. MS showed the highest overall proportion of significant proteins out of the total measured proteins, and less disparity between up and downregulated proteins.

### Gene ontology analysis highlights altered biological processes in Alzheimer’s disease

**Figure 3:**
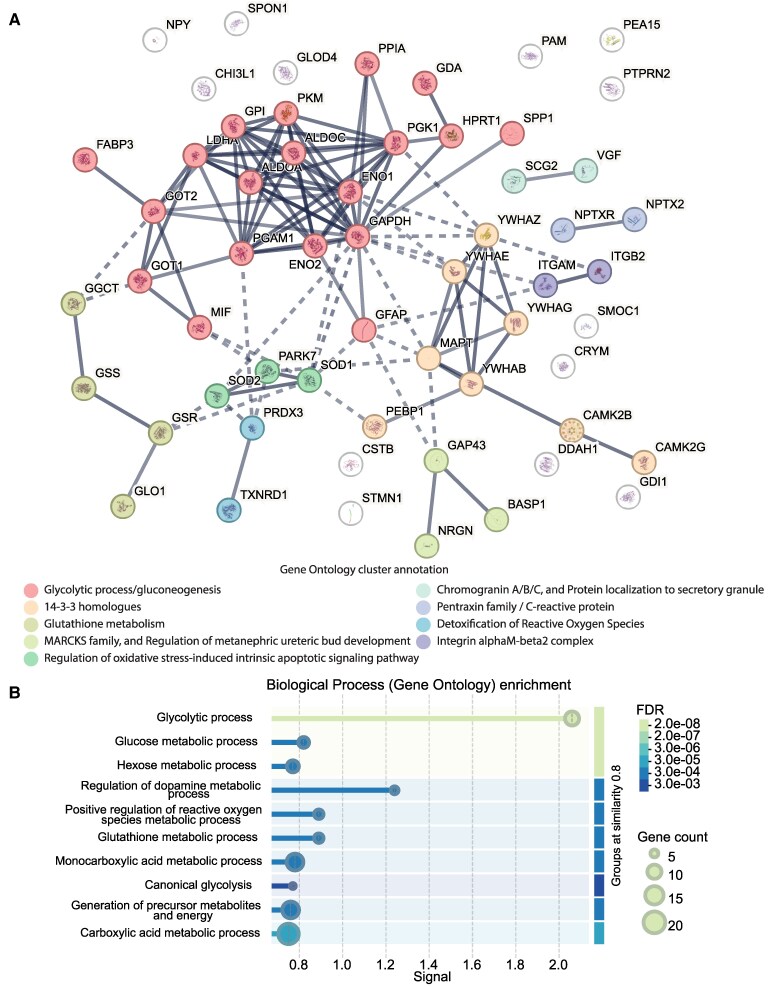
**Functional annotation of top overlapping DEPs.** (**A**) STRING network and K-means clustering of the 61 top overlapping DEPs identified 9 functional clusters, including glycolytic metabolism, 14-3-3 homologues, glutathione metabolism, apoptotic signalling, chromogranin/secretory granules, pentraxin proteins, ROS detoxification and integrin complex proteins. (**B**) GO BP enrichment analysis highlighted significant terms related to energy metabolism, neurotransmitter metabolism, oxidative stress response and cellular redox balance, including glycolytic process, glutathione metabolic process and regulation of ROS metabolism.

To determine what BPs are commonly changed across the different studies, the top 61 DEPs were used for GO analyses on the STRING-db platform. K-means clustering indicated nine functionally annotated clusters (Fig. 3A). The largest cluster of proteins was annotated to glycolytic metabolism, which included canonical glycolytic enzymes, PKM, ALDOA, ‘enolase 1 and 2’ (ENO1 and ENO2), and LDHA. Another cluster annotated to 14-3-3 homologues included four 14-3-3 proteins, YWHAB, YWHAE, YWHAG and YWHAZ. A cluster of glutathione metabolism proteins included ‘Glutathione reductase’ (GSR), ‘glutathione synthetase’ (GSS) and ‘γ-glutamyl cyclotransferase’ (GGCT). Proteins ‘superoxide dismutase 1 and 2’ (SOD1 and SOD2) and ‘Parkinson disease protein 7’ (PARK7) were annotated to a cluster related to apoptotic signalling. The remaining clusters were all defined by two functionally related proteins. ‘Secretogranin 2’ (SCG2) and VGF formed a chromogranin (CHG) and secretory granule cluster. NPTX2 and ‘Neuropentraxin receptor’ (NPTXR) formed a pentraxin cluster. ‘Thioredoxin reductase’ (TXNRD1) and ‘Thioredoxin-dependent peroxide reductase’ (PRDX3) were part of a cluster annotated to detoxification of ROS. ‘Integrin alpha-M’ (ITGAM) and ‘Integrin beta-2’ (ITGB2) were part of the integrin complex cluster. The established Alzheimer's disease associated proteins SMOC1 and CHI3L1, as well as other remaining top DEPs were not part of a functionally annotated cluster.

Enrichment of GO BP terms was assessed against a background of all proteins measured across all MS studies, revealing several significantly enriched terms (Fig. 3B). Enrichment of metabolic processes, including glycolytic process, glucose metabolic process, hexose metabolic process and canonical glycolysis, highlights altered energy metabolism in Alzheimer's disease. Additionally, processes such as regulation of dopamine metabolic process and glutathione metabolic process suggest dysregulation in neurotransmitter metabolism and oxidative stress response. Other enriched terms included positive regulation of ROS metabolic process and carboxylic acid metabolic processes, highlighting disruptions in cellular metabolism and redox balance. No enrichment of immune or inflammatory related pathways was observed amongst the top 61 DEPs. As the Olink platform contains specified immune/inflammatory panels, we explored whether overlapping proteins within Olink studies indicated enrichment for those pathways (Supplementary Fig. 3). Thirty-eight DEPs were found to overlap between at least two Olink studies and only one DEP, ‘syndecan-4’ was found in all three Olink studies, and functional enrichment analysis of these DEPs did not indicate any immune or inflammatory pathways.

### Analysis of reported fold-changes of consistently found differentially expressed proteins

**Figure 4:**
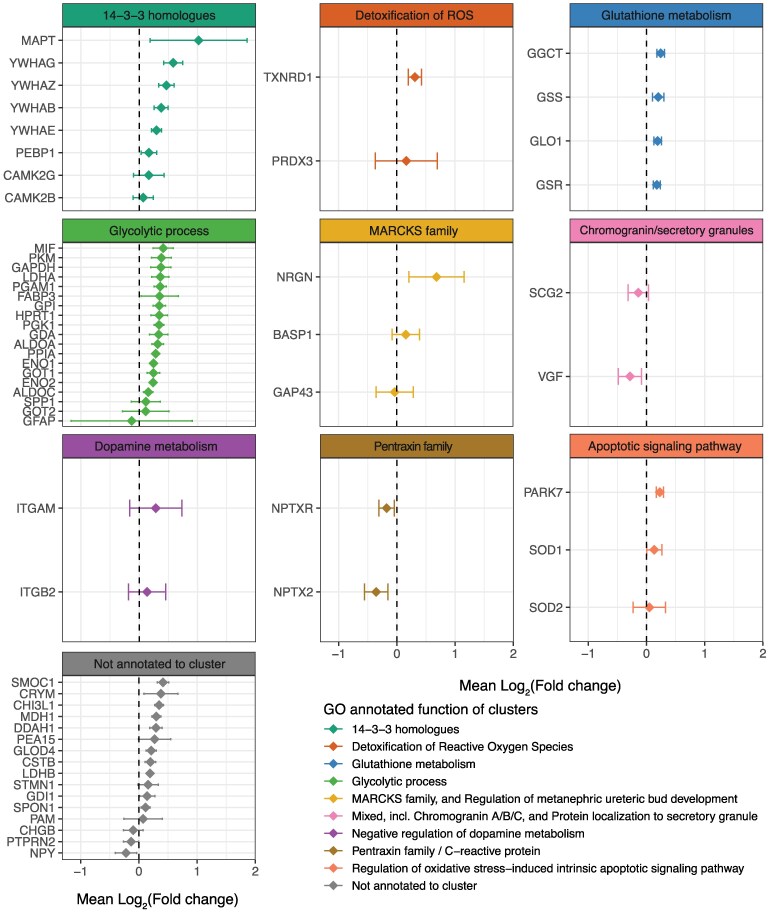
**Compilation of FCs of top DEPs.** Log2 FCs of the top DEPs were collected across all studies. Forest plots of the DEPs across the nine biological process clusters indicate, which biological functions were upregulated or downregulated in Alzheimer's disease. Markers of glycolytic metabolism, 14-3-3 homologues, and glutathione metabolism were consistently upregulated in Alzheimer's disease, whereas markers of the synaptic pentraxin family and the granin family were consistently downregulated in Alzheimer's disease. ROS, reactive oxygen species; MARCKS, myristoylated alanine-rich C kinase substrate.

We examined the reported FCs for the top DEPs. Where available, reported FCs were collected across studies, and transformed to the same log2 space. Overall, log2 FC data from 13 MS studies were compiled and the distribution of the 61 top DEPs was analysed as a forest plot with 95% CI across the pathways they were annotated to (Fig. 4; Supplementary Fig. 4; Supplementary Table 7). Five of the 61 DEPs were found to be consistently downregulated with an average FC and CI below 0. These belonged to the neuropentraxin cluster (NPTXR and NPTX2), the CHG/secretogranin cluster (SCG2 and VGF), or were not clustered to a functionally annotated group (NPY and ‘protein tyrosine phosphatase receptor type N2’). 14-3-3 proteins YWHAB, YWHAE, YWHAG and YWHAZ and the glycolytic enzymes, PKM, ALDOA, LDHA, GAPDH, PGK1 and ENO1/2 were all found to be upregulated with an average FC and CI above 0. The FC of ‘Glial fibrillary acidic protein’ (GFAP) showed a large spread with no consistent indication of upregulation or downregulation. All the markers of glutathione metabolism (GSR, GSS, GLO1 and GGCT) were consistently upregulated. The two top overlapping DEPs, SMOC1 and CHI3L1, also showed a consistent upregulation in Alzheimer's disease. Collectively, these results indicate consistent enrichment of several key biological pathways across studies and their directions of change in Alzheimer's disease pathology.

### Changes of top cerebrospinal fluid proteins over time and in brain tissue

**Figure 5:**
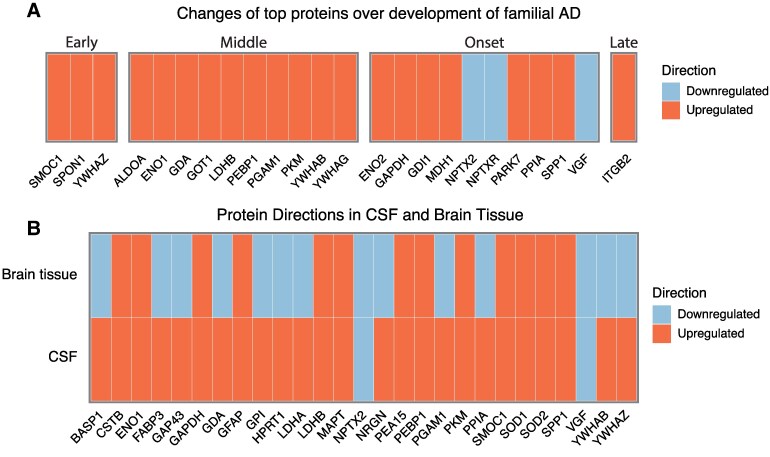
**Comparison of DEPs with familial disease onset and brain tissue datasets.** (**A**) Changes of DEPs over different phases along the disease progression of familial Alzheimer's disease as measured by Johnson *et al*.^4^ Proteins were binned into 4 stages, early (25+ years before clinical onset), mid (25–5 years before clinical onset), onset (5 years before—5 years after clinical onset) and late (5+ years after onset). All directions of change observed in our analysis agreed with the changes measured by Johnson *et al*., (2023). (**B**) Overlap of DEP changes in CSF with brain tissue data. Tissue data from the Neuropro database was compared with the top DEPs from our analysis. Only the downregulation of NPTX2 and VGF agreed between brain tissue and CSF. All other proteins that were downregulated in brain tissue were upregulated in CSF.

To further explore the role of the top overlapping DEPs in Alzheimer's disease pathology, we compared our findings with data from a study of familial Alzheimer's disease,^4^ which measured a set of Alzheimer's disease markers with targeted MS over the course of disease progression in autosomal dominant Alzheimer's disease (Fig. 5A). Twenty-four of the top DEPs were measured in the familial Alzheimer's disease study, which we then subdivided into different groups corresponding to disease stage in familial Alzheimer's disease. All directions of change measured by Johnson *et al.,* (2023)^4^ agreed with the consistent directions of change found in our analyses. Top DEPs SMOC1, ‘spondin-1’, and YWHAZ were the earliest markers to increase. During the mid-phase of disease progression, increases of DEPs included the glycolytic enzymes, ALDOA, ENO1, LDHB, ‘Phosphoglycerate mutase 1’ (PGAM1) and PKM. Other DEPs in the mid-phase that were increased include 14-3-3 proteins YWHAB and YWHAG. During disease onset, neuronal signalling proteins NPTX2, NPTXR and VGF were the only DEPs to decrease. The metabolic enzymes ENO2, GAPDH and MDH1 were also increased during onset. The only DEP that is increased after disease onset was ITGB2.

To examine the overlap of the CSF data with changes in brain tissue, we compared the top 61 DEPs with a well-curated, publicly available repository on Alzheimer's disease proteomics in brain tissue^32^ (Fig. 5B). There were 27 top DEPs found in at least 5 tissue studies. All 13 proteins that were found to be upregulated in brain tissue were also upregulated in CSF. However, only 2 of the 14 proteins, NPTX2 and VGF that were downregulated in tissue were also downregulated in CSF.

## Discussion

A deeper understanding of the heterogeneous nature of Alzheimer's disease pathology and the development of corresponding biomarkers is crucial to improve patient stratification across disease subpopulations, track disease progression and design targeted therapeutic interventions. In this study, we provide an overview of the proteins commonly found to be altered in CSF in Alzheimer's disease. Combining data from both MS-based and affinity-based studies we established a set of top DEPs that are consistently found across studies. After assessing study overlaps, we saw that most DEPs were unique to a single study. This raises the question whether these unique DEPs represent true biological signals missed by other studies, perhaps peculiar to the specific methodologies used, or are otherwise false. Recent advances in proteomics technology have enabled much deeper screenings, uncovering an increasing number of proteins overall.^19^ We showed that increased screening depth increased the number of significant DEPs found. Although no significant linear effect was observed, studies with greater depth showed more consensus in their DEPs compared to studies with lesser screening depth. More consistent and more sensitive screening techniques in future studies will determine whether these unique DEPs are reproducible.

An important consideration in determining the overlap with the MS- and affinity-based studies is biased target inclusion in the affinity-based panels. Some top DEPs we identified, like PKM, NPTXR and NPTX2, are currently not included in the Olink panels, leading to an underestimation in overlap of these proteins across all studies. A recent study performed a technological comparison of the MS, Olink and SomaScan assays indicating a good correlation in protein measurements between all platforms in CSF.^27^ However, it was also found that CSF measurements with SomaScan were close to background noise, which would limit its effectiveness in detecting low-abundance proteins, especially for proteins that decrease in Alzheimer's disease. Both MS-based and affinity-based techniques offer unique benefits and drawbacks for biomarker discovery and clinical applications. We speculate that the continued co-evolution of these methods will shape their respective roles and determine which technique is best suited for advancing research and clinical practice.

Using clustering and functional enrichment analyses on the top DEPs, we highlighted the key biological pathways that were found to be consistently altered in Alzheimer's disease. One of the main findings was the consistent upregulation of the glycolytic metabolism pathway. This was represented by upregulation of multiple glycolytic enzymes including PKM, ALDOA, PGK1, PGAM1, ENO1/2, GAPDH and GPI. Furthermore, integration with data from familial Alzheimer's disease indicated ALDOA, ENO1, PKM and PGAM1 are all upregulated in the early to middle stages of disease onset. Changes in glucose metabolism are strongly implicated in Alzheimer's disease pathology, and brain hypometabolism, as measured by brain glucose uptake with 18F-fluorodeoxyglucose-PET, is considered an early indicator of Alzheimer's disease pathology.^4,11,13,35-37^ These results point towards a crucial role of the dysregulation of glucose metabolism in the early to middle stages of Alzheimer's disease and indicate several markers as potential biomarkers and therapeutic targets.

Glutathione metabolism is the brain's main mechanism to protect against oxidative stress by neutralizing ROS and maintaining redox homeostasis.^38^ Levels of glutathione have been found to be decreased in Alzheimer's disease, but some heterogeneity in those results remain.^39-41^ Our analysis found a consistent upregulation of glutathione metabolism markers, GGCT, GSS, GLO1 and GSR. This upregulation of glutathione regulating enzymes could potentially indicate a response to increased oxidative stress in Alzheimer's disease. Recent studies have implicated a protective effect of GSR with higher levels of the protein being associated with less cognitive decline in Alzheimer's disease.^42,43^ Activation of GSS in combination with a synthetic glutathione precursor has also been shown to be protective of amyloid-ß plaque toxicity in a mouse model.^44^ Modulating glutathione metabolism as a potential therapeutic target for Alzheimer's disease remains a topic of further research, and the proteins highlighted here may represent promising candidates as biomarkers of these interventions after further validation with targeted assays in independent cohorts. Two other proteins, TXNRD1 and PRDX3, related to oxidative stress and ROS detoxification were identified in our analyses. Both been implicated in Alzheimer's disease,^45,46^ with TXNRD1 showing a consistent upregulation in Alzheimer's disease, while the changes in PRDX3 were more variable.

Multiple 14-3-3 homologues, YWHAB, YWHAE, YWHAG and YWHAZ, were also found to be consistently upregulated in Alzheimer's disease. 14-3-3 proteins are a family of conserved regulatory proteins that bind to phosphoserine-containing proteins and function as hub proteins that facilitate a wide variety of cellular processes, including signal transduction, apoptosis and cell cycle regulation.^47^ Our analysis suggest 14-3-3 proteins are changed in the early and middle stages of disease in familial Alzheimer's disease and are also found to be altered in brain tissue. Functionally, they have been shown to interact with both amyloid-ß plaques and phosphorylated tau molecules making them viable targets for therapeutic interventions.^48,49^

The only consistently reported downregulated processes in Alzheimer's disease were the neuropentraxins and granin proteins. The neuropentraxins NPTXR and NPTX2 form extracellular scaffolding proteins essential for synaptic function. The downregulation of both proteins occurs during the onset of clinical symptoms in familial Alzheimer's disease, and our analysis found that the downregulation of NPTX2 in CSF corresponds to a downregulation in brain tissue. Both proteins have been suggested as potential biomarkers for the synapse loss in Alzheimer's disease,^50-54^ and decreasing levels of NPTXR in CSF has been shown to correspond with more severe Alzheimer's disease pathology.^55^

The downregulated granin proteins, VGF and SCG2, have been suggested as biomarkers for both Alzheimer's disease and other neurodegenerative diseases.^29,56,57^ VGF and its derived peptides are secreted by neurons and regulate synaptic plasticity and neurogenesis, playing a critical role in maintaining synaptic integrity and cognitive functions.^58^ SCG2 is a precursor for the neuropeptide, secretoneurin, which has been shown to be protective against synaptic loss in the hippocampus,^59^ and against hypoxic stress after stroke.^60^ VGF, SCG2 and other granin proteins, like CHGB, are known to exist in CSF as various post-translationally modified proteoforms.^61^ In the studies reported here, and in the compilation between these studies, these different proteoforms are all mapped back to the same protein ID. Underlying differences in these specific proteoforms could have contributed to the variability in reported FCs of these proteins. Similarly, ‘microtubule associated protein tau’ (MAPT), one of the main pathological proteins of Alzheimer's disease,^62,63^ showed one of the highest positive fold changes but also a large variability. This may stem from differences in the detection and quantification of tau isoforms and phosphorylated peptides (e.g. p-tau181 and p-tau217) across studies, all of which indicate MAPT but may differ in detectability and/or abundance depending on the study.

The top 2 DEPs, SMOC1 and CHI3L1, were not clustered to any functionally annotated group of proteins. SMOC1 is a matrisomal protein that colocalizes with amyloid-ß plaques and, despite a wide association with Alzheimer's disease pathology, its function is still unclear.^34,64^ CHI3L1 is primarily expressed by astrocytes and associated with neuroinflammation and tissue remodelling in neurodegenerative diseases, including Alzheimer's disease.^65^ It has been implicated in regulating glial activation and promoting protective or reparative responses during disease progression.^33,66^ Though likely candidates, their potential as biomarkers or therapeutic targets remain a topic for further investigation.^34,65^

We were intrigued to find little representation of immune system or inflammatory proteins in our results. Alzheimer's disease pathology has been shown to have considerable immune and inflammatory related components with the activation of astrocytes and microglia.^67,68^ CHI3L1 and ‘soluble triggering receptor expressed on myeloid cells 2’ (sTREM2) have been suggested as markers of these process,^69-71^ though sTREM2 was only reported as a DEP in two studies here (Supplementary Table 3). The Olink studies reported here all included the Olink inflammatory panel, but still no enrichment of immune pathways was found when considering only the overlap between Olink studies. Although there might be considerable contribution of inflammation and immune related proteins in Alzheimer's disease pathology, we speculate that these changes might be too variable between patients to have been picked up in these large-scale studies and that targeted measurements might be able to further elucidate their role in Alzheimer's disease pathology.

There were several limitations to our analyses, highlighting potential for future studies. Firstly, collecting data from studies remained challenging. Although many studies reported their key findings, access to full datasets of complete FC information was variable. Open availability of such large-scale datasets is imperative for advancement of knowledge through the integration of results as attempted here.^72^ Furthermore, the inconsistency in FC data formats across the different proteomic platforms assessed here presented issues with direct comparison between studies. While MS datasets provided quantitative FC values, the affinity-based platforms only reported abstract scores. This inconsistency in data presentation limited our ability to perform direct FC comparisons with studies using the affinity-based methods.

Understanding the earliest biochemical changes of Alzheimer's disease, in its pre-clinical stages including pre-symptomatic and mild cognitive impairment, is critical to develop early interventions that could stem the tide of the disease.^73^ Too few studies reported data from pre-clinical stages to use in our analysis. As more large-scale proteomics studies become available, integration across the pre-clinical stages is important to define the pathological changes during Alzheimer's disease progression.

In conclusion, our analysis provides a comprehensive overview of the consistent proteomic changes reported in CSF in Alzheimer's disease, featuring key BPs driving Alzheimer's disease pathology. We highlight specific proteins linked to these processes that offer exciting opportunities for further development as biomarkers and potential therapeutic targets.

## Supplementary Material

fcaf202_Supplementary_Data

## Data Availability

All data used in this study was obtained from prior publications listed in Table 1. The data supporting the conclusions in this manuscript can be found in the Supplementary data. Underlying code is hosted on Github (github.com/ACTRU/CSF-Proteomics-Compilation).

## References

[1] Young-Pearse TL, Lee H, Hsieh YC, Chou V, Selkoe DJ. Moving beyond amyloid and tau to capture the biological heterogeneity of Alzheimer’s disease. Trends Neurosci. 2023;46(6):426–444. 37019812 10.1016/j.tins.2023.03.005 PMC10192069

[2] Duara R, Barker W. Heterogeneity in Alzheimer’s disease diagnosis and progression rates: Implications for therapeutic trials. Neurotherapeutics. 2022;19(1):8–25. 35084721 10.1007/s13311-022-01185-z PMC9130395

[3] Jack CR, Knopman DS, Jagust WJ, et al Hypothetical model of dynamic biomarkers of the Alzheimer’s pathological cascade. Lancet Neurol. 2010;9(1):119–128. 20083042 10.1016/S1474-4422(09)70299-6 PMC2819840

[4] Johnson ECB, Bian S, Haque RU, et al Cerebrospinal fluid proteomics define the natural history of autosomal dominant Alzheimer’s disease. Nat Med. 2023;29(8):1979–1988. 37550416 10.1038/s41591-023-02476-4 PMC10427428

[5] Spellman DS, Wildsmith KR, Honigberg LA, et al Development and evaluation of a multiplexed mass spectrometry based assay for measuring candidate peptide biomarkers in Alzheimer’s disease neuroimaging initiative (ADNI) CSF. Proteomics Clin Appl. 2015;9(7–8):715–731. 25676562 10.1002/prca.201400178 PMC4739636

[6] Dayon L, Núñez Galindo A, Wojcik J, et al Alzheimer disease pathology and the cerebrospinal fluid proteome. Alzheimers Res Ther. 2018;10(1):1–12. 30021611 10.1186/s13195-018-0397-4 PMC6052524

[7] Sathe G, Na CH, Renuse S, et al Quantitative proteomic profiling of cerebrospinal fluid to identify candidate biomarkers for Alzheimer’s disease. Proteomics Clin Appl. 2019;13(4):e1800105. 30578620 10.1002/prca.201800105 PMC6639119

[8] Bader JM, Geyer PE, Müller JB, et al Proteome profiling in cerebrospinal fluid reveals novel biomarkers of Alzheimer’s disease. Mol Syst Biol. 2020;16(6):e9356. 32485097 10.15252/msb.20199356 PMC7266499

[9] Higginbotham L, Ping L, Dammer EB, et al Integrated proteomics reveals brain-based cerebrospinal fluid biomarkers in asymptomatic and symptomatic Alzheimer’s disease. Sci Adv. 2020;6(43):eaaz9360. 33087358 10.1126/sciadv.aaz9360 PMC7577712

[10] Bai B, Wang X, Li Y, et al Deep multilayer brain proteomics identifies molecular networks in Alzheimer’s disease progression. Neuron. 2020;105(6):975–991.e7. 31926610 10.1016/j.neuron.2019.12.015 PMC7318843

[11] Johnson ECB, Dammer EB, Duong DM, et al Large-scale proteomic analysis of Alzheimer’s disease brain and cerebrospinal fluid reveals early changes in energy metabolism associated with microglia and astrocyte activation. Nat Med. 2020;26(5):769–780. 32284590 10.1038/s41591-020-0815-6 PMC7405761

[12] Park SA, Jung JM, Park JS, et al SWATH-MS analysis of cerebrospinal fluid to generate a robust battery of biomarkers for Alzheimer’s disease. Sci Rep. 2020;10(1):7423. 32366888 10.1038/s41598-020-64461-y PMC7198522

[13] de Geus MB, Leslie SN, Lam TK, et al Mass spectrometry in cerebrospinal fluid uncovers association of glycolysis biomarkers with Alzheimer’s disease in a large clinical sample. Sci Rep. 2023;13(1):22406. 38104170 10.1038/s41598-023-49440-3 PMC10725469

[14] Haque R, Watson CM, Liu J, et al A protein panel in cerebrospinal fluid for diagnostic and predictive assessment of Alzheimer’s disease. Sci Transl Med. 2023;15(712):eadg4122. 37672565 10.1126/scitranslmed.adg4122 PMC10880442

[15] Liu P, Li L, He F, et al Identification of candidate biomarkers of Alzheimer’s disease via multiplex cerebrospinal fluid and serum proteomics. Int J Mol Sci. 2023;24(18):14225. 37762527 10.3390/ijms241814225 PMC10532410

[16] Modeste ES, Ping L, Watson CM, et al Quantitative proteomics of cerebrospinal fluid from African Americans and caucasians reveals shared and divergent changes in Alzheimer’s disease. Mol Neurodegener. 2023;18(1):48. 37468915 10.1186/s13024-023-00638-z PMC10355042

[17] Watson CM, Dammer EB, Ping L, et al Quantitative mass spectrometry analysis of cerebrospinal fluid protein biomarkers in Alzheimer’s disease. Sci Data. 2023;10(1):261. 37160957 10.1038/s41597-023-02158-3 PMC10170100

[18] Tijms BM, Vromen EM, Mjaavatten O, et al Cerebrospinal fluid proteomics in patients with Alzheimer’s disease reveals five molecular subtypes with distinct genetic risk profiles. Nat Aging. 2024;4(1):33–47. 38195725 10.1038/s43587-023-00550-7 PMC10798889

[19] Bader JM, Albrecht V, Mann M. MS-based proteomics of body fluids: The end of the beginning. Mol Cell Proteomics. 2023;22(7):100577. 37209816 10.1016/j.mcpro.2023.100577 PMC10388585

[20] Bai B, Vanderwall D, Li Y, et al Proteomic landscape of Alzheimer’s disease: Novel insights into pathogenesis and biomarker discovery. Mol Neurodegener. 2021;16(1):55. 34384464 10.1186/s13024-021-00474-z PMC8359598

[21] del Campo M, Vermunt L, Peeters CFW, et al CSF proteome profiling reveals biomarkers to discriminate dementia with Lewy bodies from Alzheimer’s disease. Nat Commun. 2023;14(1):5635. 37704597 10.1038/s41467-023-41122-y PMC10499811

[22] Pichet Binette A, Gaiteri C, Wennström M, et al Proteomic changes in Alzheimer’s disease associated with progressive Aβ plaque and tau tangle pathologies. Nat Neurosci. 2024;27(10):1880–1891. 39187705 10.1038/s41593-024-01737-w PMC11452344

[23] Kamalian A, Ho SG, Patel M, et al Exploratory assessment of proteomic network changes in cerebrospinal fluid of mild cognitive impairment patients: A pilot study. Biomolecules. 2023;13(7):1094. 37509130 10.3390/biom13071094 PMC10377001

[24] Guo Y, Chen SD, You J, et al Multiplex cerebrospinal fluid proteomics identifies biomarkers for diagnosis and prediction of Alzheimer’s disease. Nat Hum Behav. 2024;8(10):2047–2066. 38987357 10.1038/s41562-024-01924-6

[25] Wik L, Nordberg N, Broberg J, et al Proximity extension assay in combination with next-generation sequencing for high-throughput proteome-wide analysis. Mol Cell Proteomics. 2021;20:100168. 34715355 10.1016/j.mcpro.2021.100168 PMC8633680

[26] Gold L, Ayers D, Bertino J, et al Aptamer-based multiplexed proteomic technology for biomarker discovery. PLoS One. 2010;5(12):e15004. 21165148 10.1371/journal.pone.0015004 PMC3000457

[27] Dammer EB, Ping L, Duong DM, et al Multi-platform proteomic analysis of Alzheimer’s disease cerebrospinal fluid and plasma reveals network biomarkers associated with proteostasis and the matrisome. Alzheimers Res Ther. 2022;14(1):174. 36384809 10.1186/s13195-022-01113-5 PMC9670630

[28] van Zalm PW, Ahmed S, Fatou B, et al Meta-analysis of published cerebrospinal fluid proteomics data identifies and validates metabolic enzyme panel as Alzheimer’s disease biomarkers. Cell Rep Med. 2023;4(4):101005. 37075703 10.1016/j.xcrm.2023.101005 PMC10140596

[29] Pedrero-Prieto CM, García-Carpintero S, Frontiñán-Rubio J, et al A comprehensive systematic review of CSF proteins and peptides that define Alzheimer’s disease. Clin Proteomics. 2020;17(1):21. 32518535 10.1186/s12014-020-09276-9 PMC7273668

[30] Portelius E, Brinkmalm G, Pannee J, et al Proteomic studies of cerebrospinal fluid biomarkers of Alzheimer’s disease: An update. Expert Rev Proteomics. 2017;14(11):1007–1020. 28942688 10.1080/14789450.2017.1384697

[31] Szklarczyk D, Gable AL, Lyon D, et al STRING v11: Protein-protein association networks with increased coverage, supporting functional discovery in genome-wide experimental datasets. Nucleic Acids Res. 2019;47(D1):D607–D613. 30476243 10.1093/nar/gky1131 PMC6323986

[32] Askenazi M, Kavanagh T, Pires G, Ueberheide B, Wisniewski T, Drummond E. Compilation of reported protein changes in the brain in Alzheimer's disease. Nat Commun. 2023;14(1). doi:10.1038/s41467-023-40208-x PMC10368642 37491476

[33] Connolly K, Lehoux M, O’Rourke R, et al Potential role of chitinase-3-like protein 1 (CHI3L1/YKL-40) in neurodegeneration and Alzheimer’s disease. Alzheimers Dement. 2023;19(1):9–24. 35234337 10.1002/alz.12612 PMC9437141

[34] Balcomb K, Johnston C, Kavanagh T, et al SMOC1 colocalizes with Alzheimer’s disease neuropathology and delays Aβ aggregation. Acta Neuropathol. 2024;148(1):72. 39585417 10.1007/s00401-024-02819-6 PMC11588930

[35] Paciotti S, Wojdała AL, Bellomo G, et al Potential diagnostic value of CSF metabolism-related proteins across the Alzheimer’s disease continuum. Alzheimers Res Ther. 2023;15(1):124. 37454217 10.1186/s13195-023-01269-8 PMC10350263

[36] Kumar V, Kim SH, Bishayee K. Dysfunctional glucose metabolism in Alzheimer’s disease onset and potential pharmacological interventions. Int J Mol Sci. 2022;23(17):9540. 36076944 10.3390/ijms23179540 PMC9455726

[37] Ou YN, Xu W, Li JQ, et al FDG-PET as an independent biomarker for Alzheimer’s biological diagnosis: A longitudinal study. Alzheimers Res Ther. 2019;11(1):1–11. 31253185 10.1186/s13195-019-0512-1 PMC6599313

[38] Pocernich CB, Butterfield DA. Elevation of glutathione as a therapeutic strategy in Alzheimer disease. Biochim Biophys Acta. 2012;1822(5):625–630. 22015471 10.1016/j.bbadis.2011.10.003 PMC3277671

[39] Chen JJ, Thiyagarajah M, Song J, Chen QZ, Herrmann N, Lanctôt KL. Altered central and peripheral glutathione in Alzheimer disease and mild cognitive impairment: A meta-analysis. Alzheimers Dement. 2021;17(S5):e054228.

[40] Mandal PK, Saharan S, Tripathi M, Murari G. Brain glutathione levels—A novel biomarker for mild cognitive impairment and Alzheimer’s disease. Biol Psychiatry. 2015;78(10):702–710. 26003861 10.1016/j.biopsych.2015.04.005

[41] Saharan S, Mandal PK. The emerging role of glutathione in Alzheimer’s disease. J Alzheimers Dis. 2014;40(3):519–529. 24496077 10.3233/JAD-132483

[42] Park SA, Byeon G, Jhoo JH, et al A preliminary study on the potential protective role of the antioxidative stress markers of cognitive impairment: Glutathione and glutathione reductase. Clin Psychopharmacol Neurosci. 2023;21(4):758–768. 37859449 10.9758/cpn.23.1053 PMC10591176

[43] Saul MC, Litkowski EM, Hadad N, et al Hippocampus glutathione S reductase potentially confers genetic resilience to cognitive decline in the AD-BXD mouse population. bioRxiv 2024.

[44] Raza A, Xie W, Kim KH, et al Dipeptide of ψ-GSH inhibits oxidative stress and neuroinflammation in an Alzheimer’s disease mouse model. Antioxidants. 2022;11(6):1075. 35739972 10.3390/antiox11061075 PMC9219802

[45] Xu B, Gao C, Zhang H, et al A quantitative proteomic analysis reveals the potential roles of PRDX3 in neurite outgrowth in N2a-APPswe cells. Biochem Biophys Res Commun. 2022;604:144–150. 35303681 10.1016/j.bbrc.2022.03.021

[46] Guo Y, Zhang C, Wang C, et al Thioredoxin-1 is a target to attenuate Alzheimer-like pathology in diabetic encephalopathy by alleviating endoplasmic reticulum stress and oxidative stress. Front Physiol. 2021;12:651105. 34079471 10.3389/fphys.2021.651105 PMC8166324

[47] Obsilova V, Obsil T. Structural insights into the functional roles of 14-3-3 proteins. Front Mol Biosci. 2022;9:1016071. 36188227 10.3389/fmolb.2022.1016071 PMC9523730

[48] Abdi G, Jain M, Patil N, et al 14-3-3 proteins—A moonlight protein complex with therapeutic potential in neurological disorder: In-depth review with Alzheimer’s disease. Front Mol Biosci. 2024;11:1286536. 38375509 10.3389/fmolb.2024.1286536 PMC10876095

[49] Pair FS, Yacoubian TA. 14-3-3 proteins: Novel pharmacological targets in neurodegenerative diseases. Trends Pharmacol Sci. 2021;42(4):226–238. 33518287 10.1016/j.tips.2021.01.001 PMC8011313

[50] Libiger O, Shaw LM, Watson MH, et al Longitudinal CSF proteomics identifies NPTX2 as a prognostic biomarker of Alzheimer’s disease. Alzheimers Dement. 2021;17(12):1976–1987. 33984181 10.1002/alz.12353 PMC9222372

[51] Hendrickson RC, Lee AYH, Song Q, et al High resolution discovery proteomics reveals candidate disease progression markers of Alzheimer’s disease in human cerebrospinal fluid. PLoS One. 2015;10(8):e0135365. 26270474 10.1371/journal.pone.0135365 PMC4535975

[52] Gómez de San José N, Massa F, Halbgebauer S, Oeckl P, Steinacker P, Otto M. Neuronal pentraxins as biomarkers of synaptic activity: From physiological functions to pathological changes in neurodegeneration. J Neural Transm. 2022;129(2):207–230. 34460014 10.1007/s00702-021-02411-2 PMC8866268

[53] Nilsson J, Constantinescu J, Nellgård B, et al Cerebrospinal fluid biomarkers of synaptic dysfunction are altered in Parkinson’s disease and related disorders. Mov Disord. 2023;38(2):267–277. 36504237 10.1002/mds.29287

[54] Soldan A, Oh S, Ryu T, et al NPTX2 in cerebrospinal fluid predicts the progression from normal cognition to mild cognitive impairment. Ann Neurol. 2023;94(4):620–631. 37345460 10.1002/ana.26725 PMC10543570

[55] Begcevic I, Tsolaki M, Brinc D, et al Neuronal pentraxin receptor-1 is a new cerebrospinal fluid biomarker of Alzheimer’s disease progression. F1000Res. 2018;7:1012. 30191060 10.12688/f1000research.15095.1 PMC6081984

[56] Beckmann ND, Lin WJ, Wang M, et al Multiscale causal networks identify VGF as a key regulator of Alzheimer’s disease. Nat Commun. 2020;11(1):3942. 32770063 10.1038/s41467-020-17405-z PMC7414858

[57] Duits FH, Brinkmalm G, Teunissen Charlotte E, et al Synaptic proteins in CSF as potential novel biomarkers for prognosis in prodromal Alzheimer’s disease. Alzheimers Res Ther. 2018;10(1):5. 29370833 10.1186/s13195-017-0335-x PMC6389073

[58] Quinn JP, Kandigian SE, Trombetta BA, Arnold SE, Carlyle BC. VGF as a biomarker and therapeutic target in neurodegenerative and psychiatric diseases. Brain Commun. 2021;3(4):fcab261. 34778762 10.1093/braincomms/fcab261 PMC8578498

[59] Kaufmann WA, Barnas U, Humpel C, et al Synaptic loss reflected by secretoneurin-like immunoreactivity in the human hippocampus in Alzheimer’s disease. Eur J Neurosci. 1998;10(3):1084–1094. 9753176 10.1046/j.1460-9568.1998.00121.x

[60] Shyu WC, Lin SZ, Chiang MF, et al Secretoneurin promotes neuroprotection and neuronal plasticity via the Jak2/Stat3 pathway in murine models of stroke. J Clin Invest. 2008;118(1):133–148. 18079966 10.1172/JCI32723 PMC2129236

[61] Quinn JP, Ethier EC, Novielli A, et al Cerebrospinal fluid and brain proteoforms of the granin neuropeptide family in Alzheimer’s disease. J Am Soc Mass Spectrom. 2023;34(4):649–667. 36912488 10.1021/jasms.2c00341 PMC10080684

[62] Visser PJ, Reus LM, Gobom J, et al Cerebrospinal fluid tau levels are associated with abnormal neuronal plasticity markers in Alzheimer’s disease. Mol Neurodegener. 2022;17(1):27. 35346299 10.1186/s13024-022-00521-3 PMC8962234

[63] Rawat P, Sehar U, Bisht J, Selman A, Culberson J, Reddy PH. Phosphorylated tau in Alzheimer’s disease and other tauopathies. Int J Mol Sci. 2022;23(21):12841. 36361631 10.3390/ijms232112841 PMC9654278

[64] Li-Kroeger D, Al-Ramahi I, Smith N, et al The SMOC1-matrisomal proteomics network M42 controls brain homeostasis and modulates toxicity in fly Alzheimer’s disease models. Alzheimers Dement. 2023;19(S12):e079106.

[65] Craig-Schapiro R, Perrin RJ, Roe CM, et al YKL-40: A novel prognostic fluid biomarker for preclinical Alzheimer’s disease. Biol Psychiatry. 2010;68(10):903–912. 21035623 10.1016/j.biopsych.2010.08.025 PMC3011944

[66] Yu JE, Yeo IJ, Han SB, et al Significance of chitinase-3-like protein 1 in the pathogenesis of inflammatory diseases and cancer. Exp Mol Med. 2024;56(1):1–18. 38177294 10.1038/s12276-023-01131-9 PMC10834487

[67] Jorfi M, Maaser-Hecker A, Tanzi RE. The neuroimmune axis of Alzheimer’s disease. Genome Med. 2023;15(1):6. 36703235 10.1186/s13073-023-01155-w PMC9878767

[68] Wu KM, Zhang YR, Huang YY, Dong Q, Tan L, Yu JT. The role of the immune system in Alzheimer’s disease. Ageing Res Rev. 2021;70:101409. 34273589 10.1016/j.arr.2021.101409

[69] Suárez-Calvet M, Araque Caballero MÁ, Kleinberger G, et al Early changes in CSF sTREM2 in dominantly inherited Alzheimer’s disease occur after amyloid deposition and neuronal injury. Sci Transl Med. 2016;8(369):369ra178. 10.1126/scitranslmed.aag1767 PMC5385711 27974666

[70] Shue F, White LJ, Hendrix R, et al CSF biomarkers of immune activation and Alzheimer’s disease for predicting cognitive impairment risk in the elderly. Sci Adv. 2024;10(14):eadk3674. 38569027 10.1126/sciadv.adk3674 PMC10990276

[71] Hellwig K, Kvartsberg H, Portelius E, et al Neurogranin and YKL-40: Independent markers of synaptic degeneration and neuroinflammation in Alzheimer’s disease. Alzheimers Res Ther. 2015;7(1):74. 26698298 10.1186/s13195-015-0161-y PMC4690296

[72] Shome M, MacKenzie TMG, Subbareddy SR, Snyder MP. The importance, challenges, and possible solutions for sharing proteomics data while safeguarding individuals’ privacy. Mol Cell Proteomics. 2024;23(3):100731. 38331191 10.1016/j.mcpro.2024.100731 PMC10915627

[73] Rasmussen J, Langerman H. Alzheimer’s disease—Why we need early diagnosis. Degener Neurol Neuromuscul Dis. 2019;9:123–130. 31920420 10.2147/DNND.S228939 PMC6935598

