# Supplementary material for: A compilation of reported alterations in the cerebrospinal fluid proteome in Alzheimer's disease
Source: Brain Commun. 2025 May 23;7(3):fcaf202. doi: 10.1093/braincomms/fcaf202 (PMC12146149; doi:10.1093/braincomms/fcaf202)
Supplement: fcaf202_Supplementary_Data [file fcaf202_supplementary_data.zip › Supplementary_Figures_1-4.pdf]

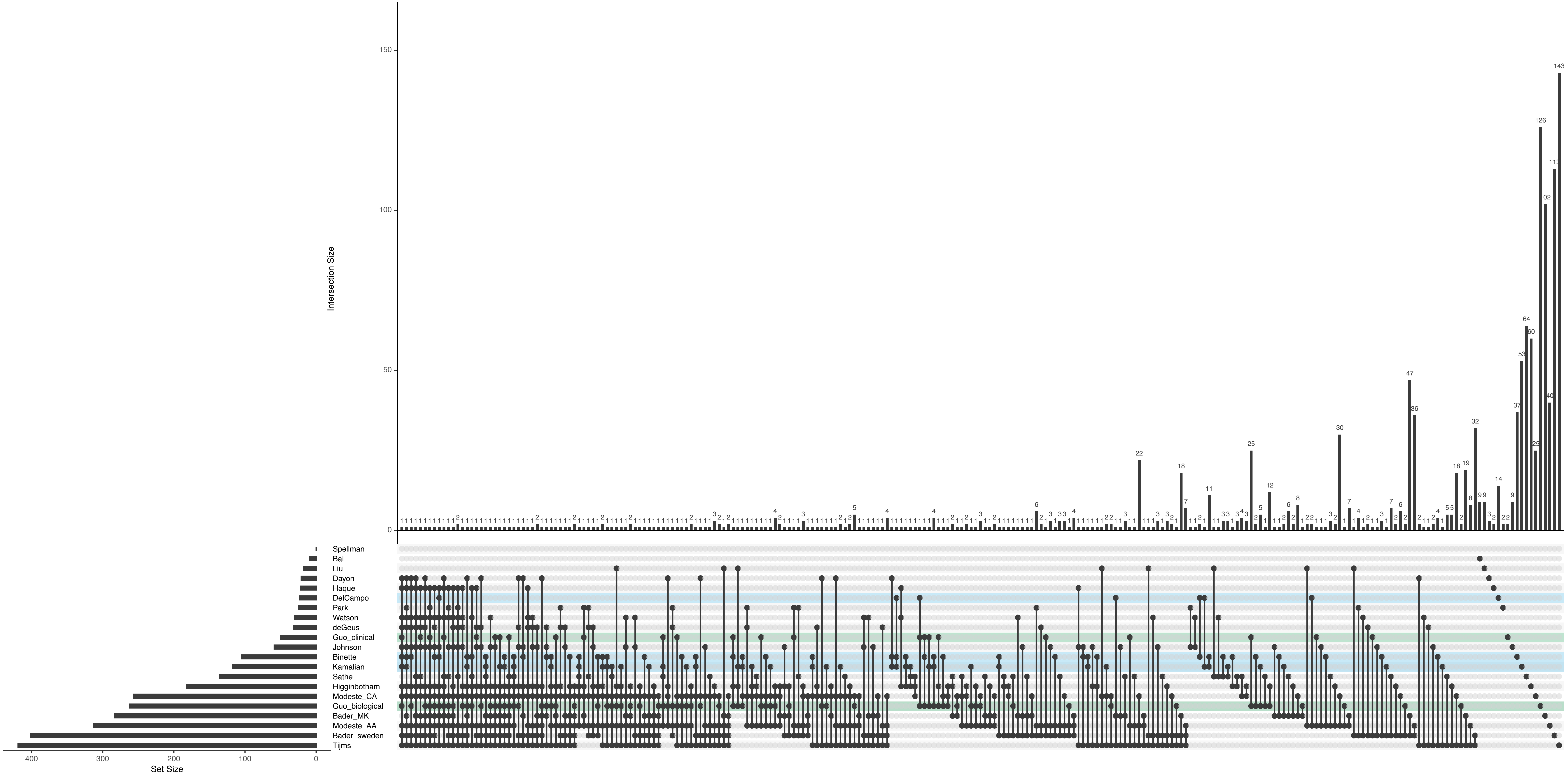

Intersection Size
150
100
50
0
Spellman
Bai
Liu
Dayon
Haque
DelCampo
Park
Watson
deGeus
Guo_clinical
Johnson
Binette
Kamalian
Sathe
Higginbotham
Modeste_CA
Guo_biological
Bader_MK
Modeste_AA
Bader_sweden
Tijms
400
300
200
100
0
Set Size

Supplementary figure 1: Full Upset plot of all significantly differentially expressed proteins (DEPs) across all studies. Specific intersections are presented on the x-axis and intersection size is shown on the y-axis. Highest overlap is shown on the left, and the number of unique proteins from all studies are shown on the right. Green highlights indicate Somalogic studies, blue highlights indicate Olink studies. Most DEPs were not replicated between studies.

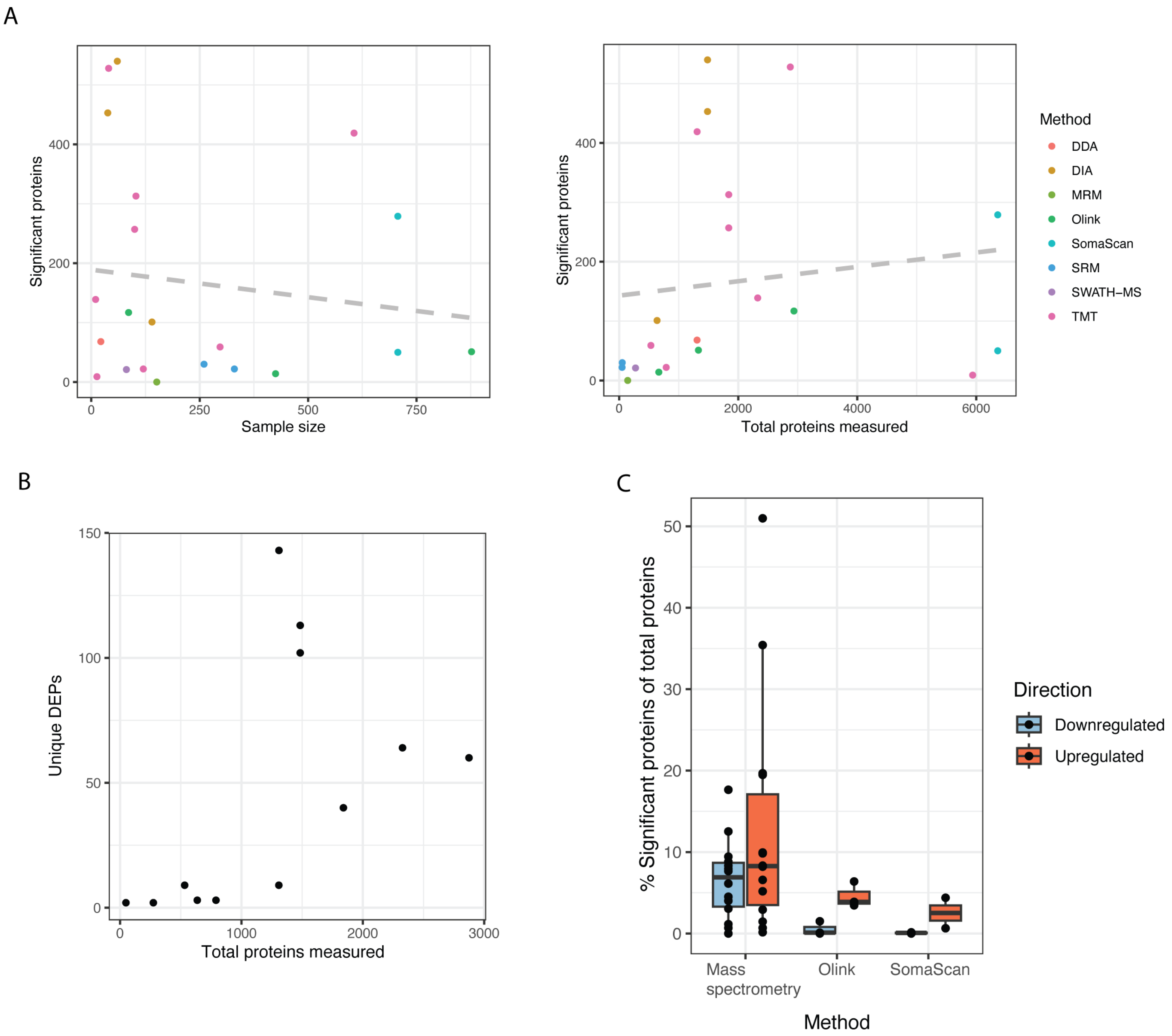

Supplementary figure 2: A) Relationship between number of significant proteins and the sample size and the total proteins measured, across all studies. No significant correlations were observed with a Pearson correlation test. Dots represent different studies and are colored by proteomic quantification method. DDA = Data-independent acquisition; DIA = Data-independent acquisition; MRM = Multiple reaction monitoring; SRM = Selected reaction monitoring; SWATH-MS = Sequential window acquisition of all theoretical mass spectra; TMT = Tandem mass tag labeled. B) Although no significant effect was observed between the screening depth of a study and the number of unique DEPs they found that had no overlap with other studies, there appeared to be a trend of increased unique DEPs with higher screening depth. C) The ratio of up- and downregulated proteins compared to the total number of proteins quantified in each study. Across the various methodologies, mass spectrometry showed the highest ratio of significant differentially expressed proteins, and overall more proteins were found upregulated than downregulated.

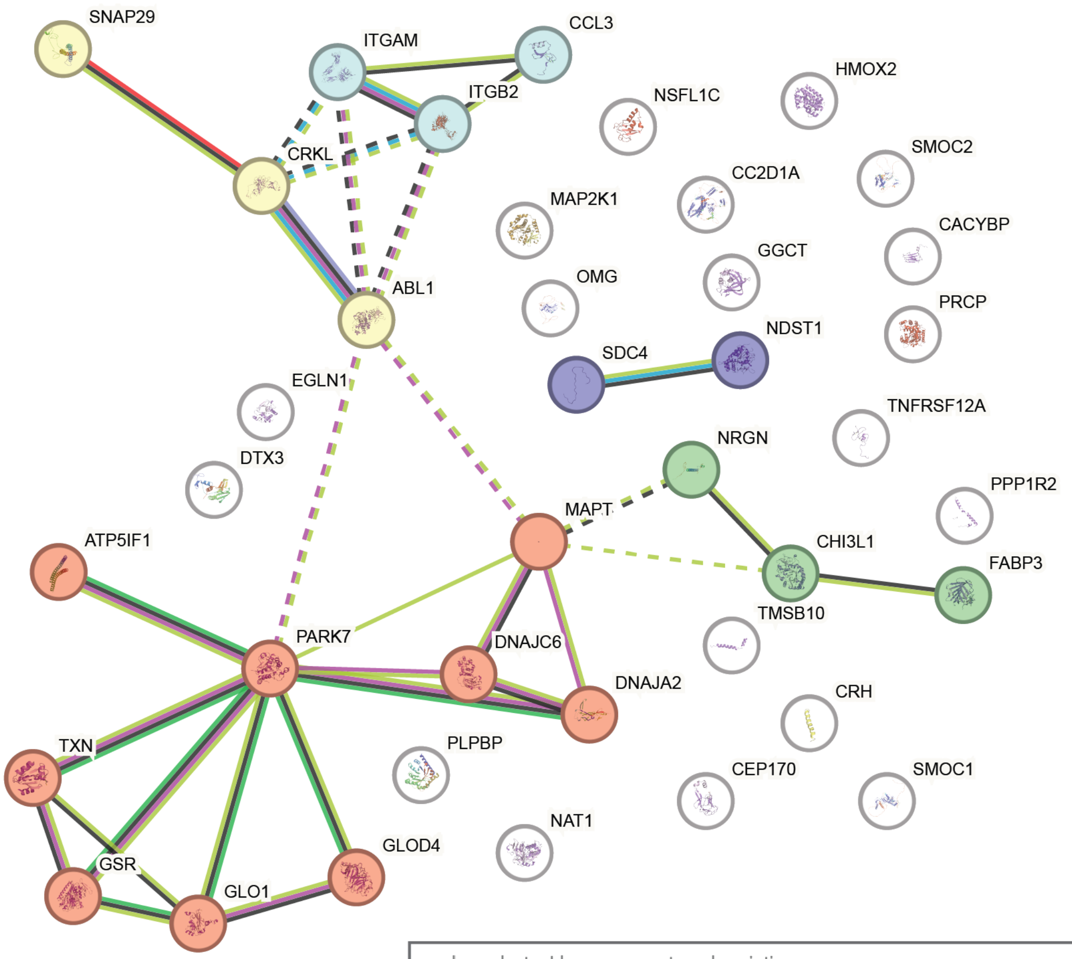

color	cluster Id	gene count	description
	Cluster 1	9	Parkinsonism
	Cluster 2	3	ABL1, CRKL, SNAP29
	Cluster 3	3	CHI3L1, FABP3, NRGN
	Cluster 4	3	+ Negative regulation of dopamine metabolism
	Cluster 5	2	HS-GAG biosynthesis

Supplementary figure 3: STRING functional network analysis of overlapping proteins measured with Olink only. A k-means clustering with 5 clusters indicated functional enrichment of pathways related to parkinsonism and regulation of dopamine metabolism.

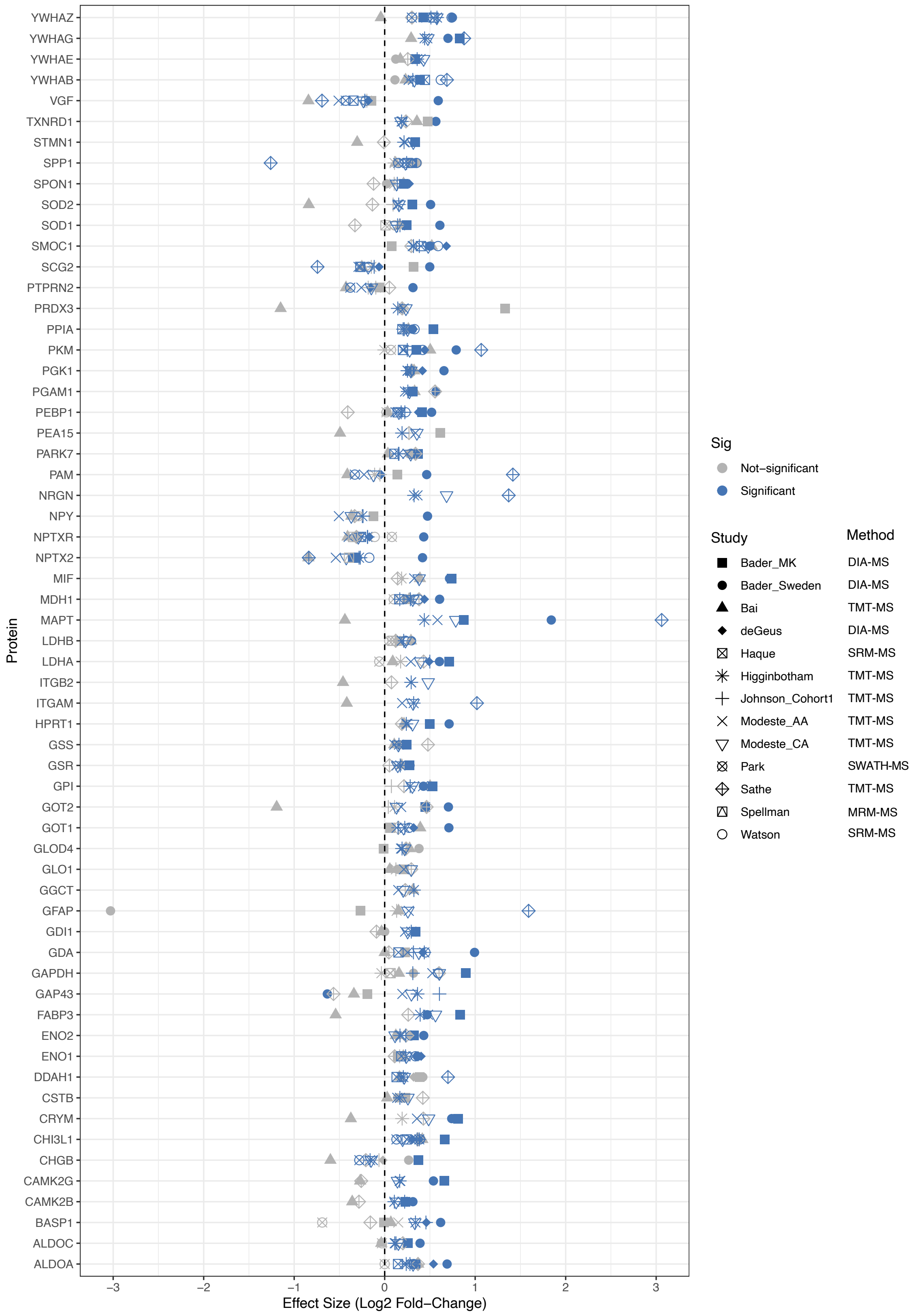

Supplementary figure 4: Reported Log2 fold-changes for the top 61 differentially expressed proteins found across all studies. Blue color indicates the protein was reported significant in that study; grey color indicates non-significant. Shapes of the points correspond to the study the proteins were measured in. Positive fold-change corresponds to upregulation in AD.
